# High-Sensitivity C-Reactive Protein Predicts Mortality and Technique Failure in Peritoneal Dialysis Patients

**DOI:** 10.1371/journal.pone.0093063

**Published:** 2014-03-25

**Authors:** Shou-Hsuan Liu, Yi-Jung Li, Hsin-Hsu Wu, Cheng-Chia Lee, Chan-Yu Lin, Cheng-Hao Weng, Yung-Chang Chen, Ming-Yang Chang, Hsiang-Hao Hsu, Ji-Tseng Fang, Cheng-Chieh Hung, Chih-Wei Yang, Ya-Chung Tian

**Affiliations:** 1 Kidney Research Center, Department of Nephrology, Lin-Kou Chang Gung Memorial Hospital and Department of Medicine, Chang Gung University, Tao Yuan, Taiwan; 2 Graduate Institute of Clinical Medical Sciences, Chang Gung University, Tao Yuan, Taiwan; University of Florida, United States of America

## Abstract

**Introduction:**

An elevated level of serum C-reactive protein (CRP) is widely considered an indicator of an underlying inflammatory disease and a long-term prognostic predictor for dialysis patients. This cross-sectional cohort study was designed to assess the correlation between the level of high-sensitivity CRP (HS-CRP) and the outcome of peritoneal dialysis (PD) patients.

**Methods:**

A total of 402 patients were stratified into 3 tertiles (lower, middle, upper) according to serum HS-CRP level and and followed up from October 2009 to September 2011. During follow-up, cardiovascular events, infection episodes, technique failure, and mortality rate were recorded.

**Results:**

During the 24-month follow-up, 119 of 402 patients (29.6%) dropped out from PD, including 28 patients (7.0%) who died, 81 patients (20.1%) who switched to hemodialysis, and 10 patients (2.5%) who underwent kidney transplantation. The results of Kaplan–Meier analysis and log-rank test demonstrated a significant difference in the cumulative patient survival rate across the 3 tertiles (the lowest rate in upper tertile). On multivariate Cox regression analysis, only higher HS-CRP level, older age, the presence of diabetes mellitus (DM), lower serum albumin level, and the occurrence of cardiovascular events during follow-up were identified as independent predictors of mortality. Every 1 mg/L increase in HS-CRP level was independently predictive of a 1.4% increase in mortality. Multivariate Cox regression analysis also showed that higher HS-CRP level, the presence of DM, lower hemoglobin level, lower serum albumin level, higher dialysate/plasma creatinine ratio, and the occurrence of infective episodes and cardiovascular events during follow-up were independent predictors of technique failure.

**Conclusions:**

The present study shows the importance of HS-CRP in the prediction of 2-year mortality and technique survival in PD patients independent of age, diabetes, hypoalbuminemia, and the occurrence of cardiovascular events.

## Introduction

An elevated level of serum C-reactive protein (CRP), an acute-phase reactant, has been found to predict the clinical outcome of various cardiovascular diseases such as myocardial infarction and stroke in the general population and in patients with chronic kidney disease and those undergoing dialysis [1], [2]. Some studies reported that CRP itself is pro-inflammatory as it has the ability to bind damaged cells and activate complements [3], [4]. A high CRP level is widely considered an indicator of an underlying inflammatory disease or a high oxidative stress condition, and a long-term prognostic predictor for patients undergoing dialysis [5]–[7]. A high-sensitivity CRP (HS-CRP) test is more sensitive than conventional CRP detection tests and can detect levels of CRP within the reference range [8].

Many studies have recognized uremic milieu as a state of chronic inflammation [9]–[12]. Even after dialysis, patients remain in an inflammatory status because the serum level of pro-inflammatory cytokines such as interleukin-1 (IL-1), IL-6, and tumor necrosis factor-α are elevated in dialysis patients [13]–[17]. It has been reported that up to 30–50% of peritoneal dialysis (PD) patients have increased CRP levels [7], [18], [19]. Although several studies have reported that the elevation of CRP is a useful predictor of the occurrence of cardiovascular events and mortality [7], [13], [20], [21], some demonstrate that CRP is not significantly associated with all-cause mortality [19], [22], [23]. It has been reported that the characteristics of membrane transporter status and residual renal function affect the serum CRP level [24], [25]. Therefore, this discrepancy may be attributed to the sample size, analyzed parameters, and study period.

In addition to predicting mortality, an elevated CRP level has been shown to be linked to technique failure. Nevertheless, only a few studies have assessed the impact of baseline serum CRP level on technique failure, and the association between baseline serum CRP level and subsequent technique failure is still inconclusive. Some studies demonstrated that serum CRP level was independently associated with technique failure [26], whereas others found that serum CRP level was not a predictor for technique failure [27], [28]. Therefore, it would be interesting to know whether serum CRP level is correlated with technique failure in PD patients.

Both residual renal function and peritoneal clearance have been reported to be inversely associated with serum CRP level [24], [25], whereas some studies demonstrate that serum CRP is not influenced by peritoneal solute transport rate and residual renal function [22], [29]. As these 2 factors have also been shown to be predictive markers for mortality and technique failure in PD patients and are associated with CRP, it would be crucial to elucidate whether HS-CRP can predict the clinical outcome in PD patients independently of residual renal function and peritoneal clearance.

The aim of this cross-sectional cohort study was to investigate whether serum HS-CRP level was independently associated with mortality and technique failure in PD patients.

## Materials and Methods

This cross-sectional cohort study complied with the guidelines of the Declaration of Helsinki and approved by the Medical Ethics Committee of Chang Gung Memorial Hospital, a tertiary referral center located in the northern part of Taiwan. Since this study involved retrospective review of existing data, the Institutional Review Board approval was obtained, but without specific informed consent from patients. In addition, all individual information was securely protected (by delinking identifying information from main data set) and available to investigators only. Furthermore, all the data were analyzed anonymously. The Institutional Review Board of Chang Gung Memorial Hospital has approved this study and no informed consent was requested by the Committee. Finally, all primary data were collected according to strengthening the reporting of observational studies in epidemiology guidelines.

### Study Population and Follow-up

This cohort observational study was conducted in the PD unit of Linkou Chang-Gung Memorial Hospital from October 2009 to September 2011 to determine the impact of HS-CRP on the patients’ outcome. All the data were obtained from the record of the routine examination. Among 445 patients, 43 patients were excluded owing to recent episodes of peritonitis, active infection, chronic liver disease, autoimmune disease, malignant diseases, or acute myocardial infarction within 3 months. The remaining 402 patients were enrolled in this study. All patients had been undergoing PD for >3 months and were observed during a period of 24 months. Patients were categorized into 3 tertiles according to the baseline HS-CRP level in October 2009.

Baseline demographic and clinical data, including age, sex, body mass index (BMI), presence of diabetes, duration of PD at the entry of this study, residual renal function, hemogram, and biochemical parameters, were recorded. PD membrane characteristics, including the dialysate/plasma creatinine ratio (D/P_Cr_) and peritoneal equilibrium test (PET) results, were assessed. All of these data were obtained during routine clinical practice. During the 24-month follow-up, cardiovascular events, infection episodes, technique failure, and mortality rate were recorded. A cardiovascular event was defined as acute myocardial infarction, intervention of coronary artery disease with angioplasty or stenting, cerebral vascular accident, and peripheral vascular occlusion requiring intervention. An infection episode was defined as PD-related infection or non-PD-related infection.

No patient was lost to follow-up. Patient deaths were recorded, and those patients who underwent kidney transplantation or were transferred to hemodialysis were censored when they received alterative renal replacement therapy.

### Laboratory Parameters

Blood specimens were collected within a few days of clinical examination during stable PD routine examination to minimize the effect of any acute event. Blood was drawn immediately, centrifuged, and then stored at −70°C until used in assays. Serum HS-CRP was analyzed by using immunonephelometry (Nanopia CRP; Daiichi, Tokyo, Japan). Serum intact parathyroid hormone (iPTH) was determined by using a chemiluminometric immunoassay (ADVIA Centaur iPTH; Siemens Medical Solutions Diagnostics, New York, NY, USA) with a reference range of 7–53 pg/mL. All other biochemical parameters were obtained with standard laboratory procedures by using an automatic analyzer.

### Peritoneal Dialysis Membrane Characteristics

The adequacy of dialysis was determined by measuring the total weekly creatinine clearance (CCr), which was normalized to 1.73 m^2^ of the body surface area. Residual renal CCr was calculated as an average of the 24-h urine urea and CCr. Dialysate creatinine concentration was corrected for interference by glucose. The dialysate/plasma creatinine ratio (D/P_Cr_) was calculated from the concentrations of creatinine in the 24-h dialysate and the plasma.

### Statistical Analysis

Continuous variables are expressed as means and standard deviations, and categorical variables as numbers and percentages in brackets. All data were tested for normality of distribution and equality of standard deviations before analysis. For comparisons between patient groups, one-way ANOVA was used for quantitative variables, whereas chi-square or Fisher’s exact test was used for categorical variables. P values were calculated, and the null hypothesis was rejected if the P value was <0.05. The independent links between HS-CRP and variables were analyzed further with simple and stepwise backward multiple linear regression analyses, adjusting for other factors linked with HS-CRP. Mortality was examined by using Kaplan–Meier analysis. Differences in the survival curves among the 3 groups were evaluated by using the log-rank test. An initial univariate Cox regression analysis was performed to compare the frequency of possible risk factors associated with mortality. To control for possible confounding factors, a multivariate Cox regression analysis (stepwise backward approach) was performed to analyze the significant factors (P<0.05) on the univariate analysis that met the assumptions of a proportional hazard model. The criterion for significance was a 95% confidence interval to reject the null hypothesis (P<0.05). All analyses were performed by using SPSS 12.0 for Windows (SPSS Inc., Chicago, IL, USA).

## Results

### Baseline Patient Characteristics of Demography and Biochemistry

Of the 402 patients in this cohort observational study, 148 (36.8%) were men and 254 (63.2%) were women. Their mean age was 48.6±14.6 years. The mean duration of PD was 73.6±41.0 months. Table 1 shows the baseline characteristics and laboratory parameters of these 402 patients stratified into 3 tertiles according to the serum HS-CRP level. The mean HS-CRP value in the study patients was 9.57 mg/L. The mean HS-CRP values were 0.90 mg/L (0–1.77 mg/L) in the lower tertile (T1), 3.90 mg/L (1.77–7.51 mg/L) in the middle tertile (T2), and 23.90 mg/L (7.51–45.85 mg/L) in the upper tertile (T3). Among the 3 tertiles, there was no significant difference in sex and PD duration. There was a significant increase (P<0.001) in the mean age across HS-CRP tertiles: 44.3 years in T1, 49.1 years in T2, and 52.4 years in T3. The percentages of patients with diabetes were significantly increased: 8.2% in T1, 17.2% in T2, and 27.6% in T3. A significant increase in the BMI values was also found across the 3 tertiles. Up to 76.9% of patients in T1 had residual renal function, whereas 59.7% of patients in T2 and 53.0% of patients in T3 had residual renal function (P<0.001). The white blood cell count was significantly increased and the hemoglobin level was reduced, whereas the platelet count was not different across these groups. Liver biochemistry test showed no difference in serum aspartate transaminase, alanine transaminase, and total bilirubin levels across the HS-CRP tertiles. The serum albumin level was in inverse proportion to the HS-CRP level, with the lowest level in T3. The examination of lipid profiles revealed a significant decrease in high-density lipoprotein (HDL) levels and an increase in triglyceride levels across the 3 tertiles of increasing HS-CRP, whereas total cholesterol and low-density lipoprotein (LDL) levels were not different across these tertiles. Fasting blood sugar and glycated hemoglobin (HbA1c) levels were examined in every patient, and the results showed a significant increase in these 2 parameters across the 3 tertiles of increasing HS-CRP. Surprisingly, further analysis revealed that in patients without diabetes, fasting blood sugar and HbA1c levels were increased across the 3 tertiles of increasing HS-CRP, whereas in patients with diabetes, blood sugar and HbA1c levels varied and were not different across the HS-CRP tertiles. Compared with patients in the other tertiles, the serum creatinine level was lowest in the patients in T3. Serum uric acid, calcium, phosphorus, and iPTH levels were not significantly different across the HS-CRP tertiles. Transferrin saturation was significantly decreased across the 3 tertiles of increasing HS-CRP. There was a trend toward the higher ferritin level in the upper tertiles, although it did not reach statistical significance.

**Table 1:** Demographic and laboratory characteristics of the PD patients stratified according to serum HS-CRP levels.

	Total	Lower tertile	Middle tertile	Upper tertile	
	(n = 402)	(n* = *134)	(n = 134)	(n = 134)	
HS-CRP (mg/L)	9.57±16.35	0.90±0.43	3.90±1.69	23.90±22.09	P
		(<1.77)	(1.77–7.51)	(>7.51)	
Male	148 (36.8%)	43 (32.1%)	53 (39.6%)	52 (38.8%)	0.38
Age (years)	48.6±14.6	44.3±15.3	49.1±13.0	52.4±14.3	<0.001
Body mass index (kg/m^2^)	22.4±3.6	21.1±2.9	22.8±3.6	23.4±3.9	<0.001
Diabetes mellitus	71 (17.7%)	11 (8.2%)	23 (17.2%)	37 (27.6%)	<0.001
PD duration (months)	73.6±41.0	66.9±35.7	76.1±40.4	77.8±45.8	0.07
Residual urine	254 (63.2%)	103 (76.9%)	80 (59.7%)	71 (53.0%)	<0.001
WBC count (1000/μL)	7.655±2.523	6.400±1.959	7.679±2.058	8.866±2.840	<0.001
Hemoglobin (g/dL)	10.06±1.56	10.24±1.56	10.18±1.45	9.66±1.61	<0.001
Platelet count (1000/μL)	257.81±82.63	247.65±79.33	258.69±77.09	266.92±90.35	0.17
AST (U/L)	20.1±11.9	20.3±12.6	19.4±9.3	20.6±13.4	0.73
ALT (U/L)	20.2±18.4	20.3±20.5	19.9±12.2	20.5±21.2	0.9
Total bilirubin (mg/dL)	0.30±0.12	0.28±0.13	0.36±0.12	0.25±0.08	0.20
Albumin (g/dL)	3.96±0.46	4.07±0.38	3.98±0.43	3.85±0.55	<0.001
Total cholesterol (mg/dL)	202.3±49.0	205.6±44.6	203.2±49.5	198.3±52.5	0.48
HDL (mg/dL)	47.4±15.1	55.2±16.0	45.0±13.4	40.9±11.5	<0.001
LDL (mg/dL)	119.7±40.8	121.6±40.6	119.2±41.4	117.9±40.8	0.82
Triglyceride (mg/dL)	190.3±129.3	149.3±79.4	195.4±132.8	224.6±152.5	<0.001
Total patients					
Fasting sugar (mg/dL)	114.7±53.4	103.5±43.7	110.6±40.3	129.9±68.6	<0.001
HbA1c %	5.66±1.03	5.43±0.85	5.61±0.89	5.96±1.23	<0.001
Diabetic patients					
Fasting sugar (mg/dL)	187.4±90.1	194.9±114.9	172.9±61.6	193.8±97.4	0.67
HbA1c %	7.31±1.50	7.81±1.34	7.02±1.36	7.33±1.61	0.40
Non-diabetic patients					
Fasting sugar (mg/dL)	98.8±17.8	94.9±10.2	98.1±16.7	104.5±24.3	<0.001
HbA1c %	5.33±0.38	5.22±0.36	5.35±0.42	5.42±0.33	<0.001
Creatinine (mg/dL)	11.32±3.01	11.73±3.23	11.40±2.83	10.82±2.89	0.046
Uric acid (mg/dL)	6.81±1.32	6.76±1.25	6.73±1.24	6.95±1.45	0.35
Calcium (mg/dL)	10.01±1.00	9.96±1.00	10.12±0.98	9.95±0.99	0.30
Phosphorus (mg/dL)	4.94±1.32	5.09±1.28	4.90±1.27	4.83±1.42	0.26
iPTH (pg/mL)	329.0±388.8	332.8±391.3	285.8±318.3	367.2±443.4	0.25
Transferrin saturation	0.272±0.127	0.295±0.118	0.276±0.134	0.244±0.122	0.01
Ferritin (μg/L)	329.1±510.9	261.9±435.2	371.9±667.9	352.6±382.4	0.20

Note:

Continuous variables given as mean ± standard deviation, and categorical variables, as number (percentage).

Abbreviations: HS-CRP, high-sensitivity C-reactive protein; PD, peritoneal dialysis; WBC, white blood cell; AST, aspartate transaminase; ALT, alanine transaminase; HDL, high-density lipoprotein; LDL, low-density lipoprotein; HbA1c, glycated hemoglobin; iPTH, intact parathyroid hormone.

### Baseline Peritoneal Membrane Characteristics

Table 2 outlines the peritoneal membrane characteristics of the PD patients. Among the 3 tertiles of increasing HS-CRP, the value of D/P_Cr_ was significantly increased. According to the results of PET, the peritoneal transport status in the 3 tertiles was significantly different, as 12.7% of patients in T3 were high transporters and 8.2% of patients were low transporters, whereas 3.7% of patients in T1 were high transporters and 16.4% of patients were low transporters. There was no significant difference for the weekly CCr (total) in the 3 tertiles, whereas the weekly CCr (PD) in T1 was significantly lower than that in the other 2 tertiles (P<0.001). There was also a trend toward a lower value of weekly CCr (renal) in T3.

**Table 2:** Peritoneal membrane characteristics of the PD patients stratified according to HS-CRP levels.

	Total	Lower tertile	Middle tertile	Upper tertile	
	(n = 402)	(n* = *134)	(n = 134)	(n = 134)	
HS-CRP (mg/L)	9.57±16.35	0.90±0.43	3.90±1.69	23.90±22.09	P
		(<1.77)	(1.77–7.51)	(>7.51)	
Dialysate/plasma creatinine	0.64±0.12	0.62±0.12	0.65±0.10	0.66±0.13	0.01
Peritoneal equilibration test					<0.001
High	28 (7.0%)	5 (3.7%)	6 (4.5%)	17 (12.7%)	
High average	152 (37.8%)	41 (30.6%)	63 (47.0%)	48 (35.8%)	
Low average	179 (44.5%)	66 (49.3%)	55 (41.0%)	58 (43.3%)	
Low	43 (10.7%)	22 (16.4%)	10 (7.5%)	11 (8.2%)	
Weekly CCr (normalized)	59.6±13.9	58.8±13.8	60.3±14.9	59.7±13.1	0.70
Weekly CCr (total)	54.1±14.5	52.0±14.6	55.5±14.9	54.7±13.9	0.12
Weekly CCr (PD)	45.1±12.1	41.0±12.0	47.1±10.6	47.3±12.6	<0.001
Weekly CCr (renal)	8.9±15.2	11.0±13.9	8.2±16.8	7.4±14.6	0.12

Note:

Continuous variables given as mean ± standard deviation, and categorical variables, as number (percentage).

Abbreviations: PD, peritoneal dialysis; HS-CRP, high-sensitivity C-reactive protein; CCr, creatinine clearance.

### Patient Survival, Technique Survival, Cardiovascular Events, and Infection Episodes during the 24-month Follow-up

During the 24-month follow-up, 119 of 402 patients (29.6%) dropped out from PD, including 28 patients who died (7.0%), 81 patients (20.1%) who switched to hemodialysis, and 10 patients (2.5%) who underwent kidney transplantation (Table 3). There were significant differences in the mortality and technique failure in the three tertiles, whereas there was no significant difference in the number of patients transferring to HD and receiving transplantation among three groups (Table 3).

**Table 3:** Clinical outcomes in the PD patients stratified according to HS-CRP levels.

	Total	Lower tertile	Middle tertile	Upper tertile	
	(n = 402)	(n* = *134)	(n = 134)	(n = 134)	
HS-CRP (mg/L)	9.57±16.35	0.90±0.43	3.90±1.69	23.90±22.09	P
		(<1.77)	(1.77–7.51)	(>7.51)	
Outcomes					
Death	28 (7.0%)	0 (0.0%)	4 (3.0%)	24 (17.9%)	<0.001
Transfer to HD	81 (20.1%)	25 (18.7%)	25 (18.7%)	31 (23.1%)	0.575
Transplantation	10 (2.5%)	3 (2.2%)	4 (3.0%)	3 (2.2%)	0.903
Technique survival	283 (70.4%)	106 (79.1%)	101 (75.4%)	76 (56.7%)	<0.001
Infection episodes	192 (47.8%)	55 (41.0%)	66 (49.3%)	71 (53.0%)	0.14
Cardiovascular events	72 (17.9%)	17 (12.7%)	21 (15.7%)	34 (25.4%)	0.02

Note:

Continuous variables given as mean ± standard deviation, and categorical variables, as number (percentage).

Abbreviations: PD, peritoneal dialysis; HS-CRP, high-sensitivity C-reactive protein; HD, hemodialysis.

**Figure 1:**
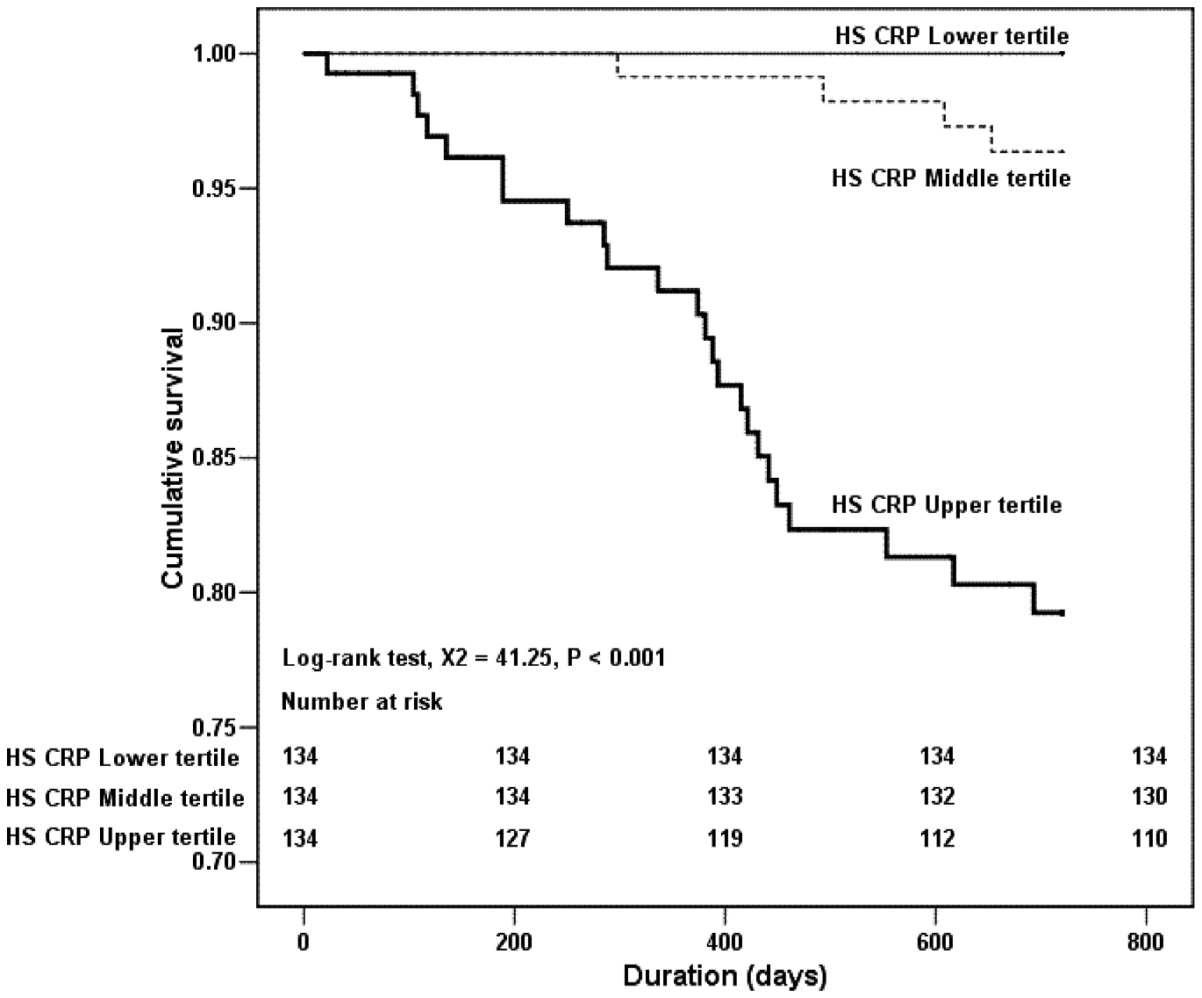
Kaplan–Meier survival curves based on serum HS-CRP levels among PD patients in the 2-year follow-up.

**Figure 2:**
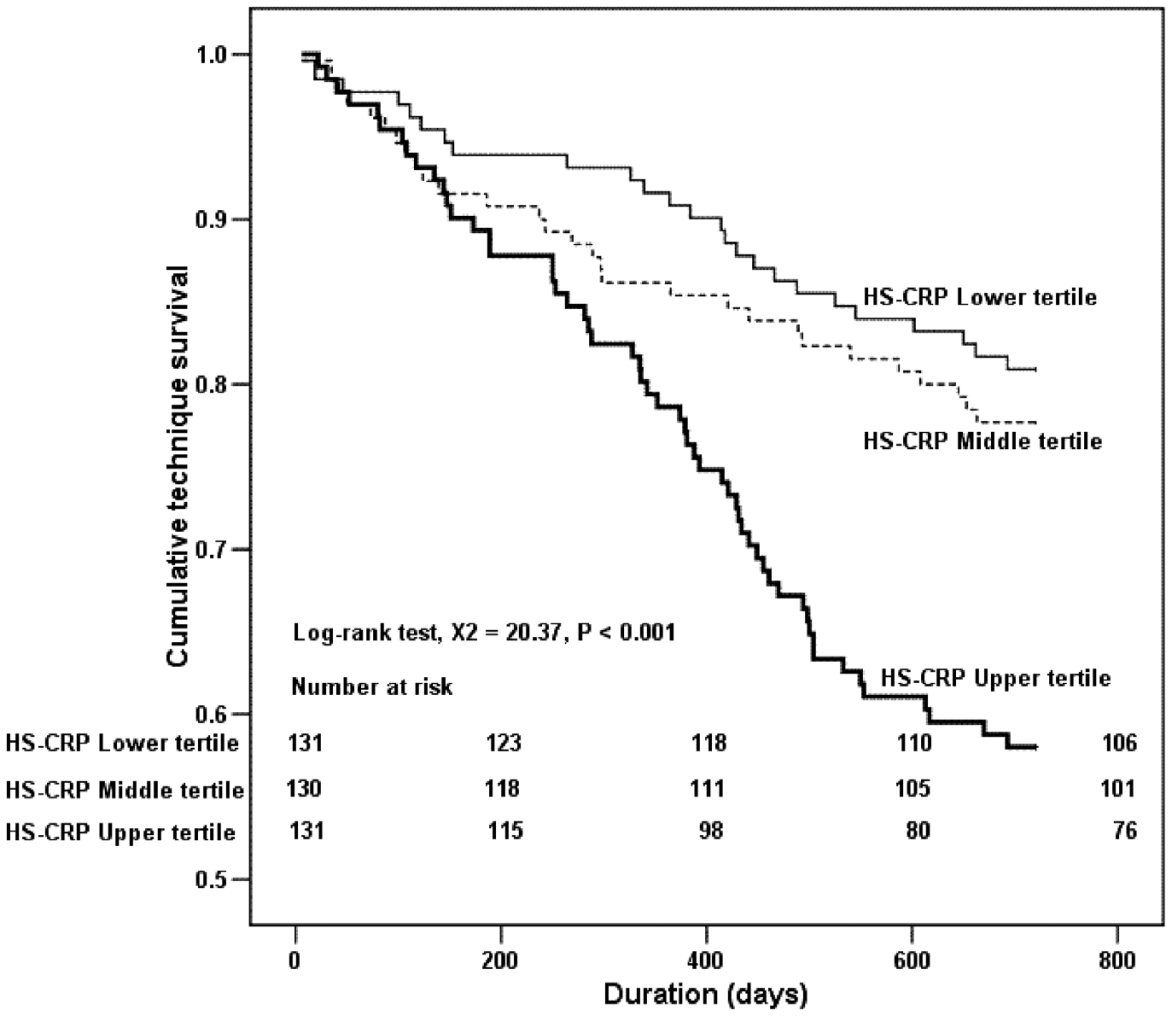
Kaplan–Meier technique survival (transplantation censored) curves based on serum HS-CRP levels among PD patients in the 2-year follow-up.

The results of the Kaplan–Meier analysis and log-rank test also demonstrated that there was significant difference in the cumulative patient survival rate between T3 vs T1 (P<0.001) and T3 vs T2 (P<0.001) (Fig. 1). Nevertheless, despite a trend towards a higher patient survival rate in T1 than that in T2, the difference did not reach statistically significant. In addition, the Kaplan–Meier analysis and log-rank test also revealed that the cumulative technique survival rate in T3 was significantly lower than T1 and T2 (T3 *vs.* T1, P<0.001; T3 *vs.* T2, P<0.001) (Fig. 2), whereas the comparison of the cumulative technique survival rate between T1 and T2 was not statistically different.

During the 24-month follow-up, there was no difference in the episodes of PD-related and non-PD-related infection across the 3 tertiles. Because serum CRP level has been identified as a cardiovascular factor, we examined the predictive value of serum HS-CRP level for subsequent cardiovascular events during the 24-month follow-up. More patients (25.4%) in T3 had cardiovascular events during the follow-up period than patients in T2 (15.7%) and T1 (12.7%) (P = 0.018). These results suggest that patients in the high HS-CRP tertiles are at risk of developing cardiovascular events in the following 24 months.

### HS-CRP Level is an Independent Predictor of Mortality and Technique Failure

To further assess the independent predictors of mortality, Cox regression analysis was used. The results of univariate Cox regression analysis revealed that higher HS-CRP level, older age, the presence of DM, higher white blood cell count, lower serum albumin level, higher plasma sugar level and HbA1c value, lower serum creatinine level, lower calcium and phosphorus levels, and the occurrence of infection episodes and cardiovascular events during the 24-month follow-up were risk factors for mortality (Table 4). On multivariate Cox regression analysis, only higher HS-CRP level, older age, the presence of DM, lower serum albumin level, and the occurrence of cardiovascular events during follow-up were identified as independent predictors of mortality (Table 5). Every 1 mg/L increase in HS-CRP level was independently predictive of a 1.4% increase in mortality.

**Table 4:** Relative risk of mortality in univariate Cox regression analysis.

		Univariate analysis	
	RR	HR (95% CI)	P
HS-CRP (mg/L)	1.025	1.014–1.035	<0.001
Male	1.692	0.719–3.981	0.23
Age	1.067	1.036–1.098	<0.001
Body mass index (kg/m^2^)	1.051	0.954–1.158	0.32
Diabetes mellitus	5.979	2.846–12.560	<0.001
PD duration (months)	0.993	0.982–1.004	0.20
Residual urine	0.700	0.331–1.480	0.35
White blood cell count (1000/μL)	1.158	1.046–1.281	0.01
Hemoglobin (g/dL)	0.859	0.684–1.079	0.19
Platelet count (1000/μL)	1.000	0.996–1.005	0.9
AST (U/L)	1.018	0.996–1.041	0.12
ALT (U/L)	1.003	0.983–1.023	0.80
Total bilirubin (mg/dL)	0.168	0.000–231.652	0.67
Albumin (g/dL)	0.194	0.110–0.341	<0.001
Total cholesterol (mg/dL)	0.996	0.988–1.004	0.31
HDL (mg/dL)	1.002	0.964–1.042	0.9
LDL (mg/dL)	0.991	0.974–1.008	0.28
Triglyceride (mg/dL)	1.001	0.999–1.004	0.34
Sugar (mg/dL)	1.008	1.004–1.012	<0.001
HbA1c %	1.532	1.232–1.905	<0.001
Creatinine (mg/dL)	0.730	0.620–0.858	<0.001
Uric acid (mg/dL)	0.746	0.541–1.028	0.07
Calcium (mg/dL)	0.653	0.476–0.896	0.01
Phosphorus (mg/dL)	0.674	0.488–0.931	0.02
iPTH (pg/mL)	0.999	0.998–1.000	0.11
Transferrin saturation	3.398	0.259–44.651	0.35
Ferritin (μg/L)	1.000	1.000–1.001	0.19
Dialysate/plasma creatinine	19.269	0.942–394.309	0.06
Peritoneal equilibration test (%)	1.612	0.995–2.613	0.05
Weekly CCr (normalized)	0.996	0.969–1.024	0.77
Weekly CCr (total)	0.992	0.966–1.018	0.54
Weekly CCr (PD)	1.003	0.973–1.034	0.85
Weekly CCr (renal)	0.990	0.961–1.019	0.49
Infection episodes	2.434	1.122–5.279	0.02
Cardiovascular events	5.979	2.847–12.557	<0.001

Note:

Abbreviations: RR, relative risk; HR, hazard ratio; CI, confidence interval; HS-CRP, high-sensitivity C-reactive protein; PD, peritoneal dialysis; AST, aspartate transaminase; ALT, alanine transaminase; HDL, high-density lipoprotein; LDL, low-density lipoprotein; HbA1c, glycated hemoglobin; iPTH, intact parathyroid hormone; CCr, creatinine clearance.

**Table 5:** Relative risk of mortality in multivariate Cox regression analysis.

		Multivariate analysis	
	RR	HR (95% CI)	P
HS-CRP (mg/L)	1.014	1.003–1.026	0.02
Age	1.042	1.011–1.074	0.01
Diabetes mellitus	3.381	1.578–7.243	0.002
Albumin (g/dL)	0.289	0.156–0.535	<0.001
Cardiovascular events	2.839	1.309–6.160	0.008

Note:

Abbreviations: RR, relative risk; HR, hazard ratio; CI, confidence interval; HS-CRP, high-sensitivity C-reactive protein.

To identify the risk factors for technique failure (death or transfer to hemodialysis), univariate and multivariate Cox regression analyses were applied. The results of univariate Cox regression analysis demonstrated that higher HS-CRP level, older age, the presence of DM, the existence of residual urine, higher white blood cell count, lower hemoglobin level, lower serum albumin level, higher plasma sugar level and HbA1c value, lower serum creatinine level, lower serum phosphorus levels, higher dialysate/plasma creatinine ratio, peritoneal equilibration test, higher weekly CCr (PD), and the occurrence of infection episodes and cardiovascular events during follow-up were significant risk factors for technique failure (Table 6). Multivariate Cox regression analysis showed that higher HS-CRP level, the presence of DM, lower hemoglobin level, lower serum albumin level, higher dialysate/plasma creatinine ratio, and the occurrence of infection episodes and cardiovascular events during follow-up were independent predictors of technique failure (Table 7).

**Table 6:** Relative risk of technique failure in univariate Cox regression analysis.

		Univariate analysis	
	RR	HR (95% CI)	P
HS-CRP (mg/L)	1.016	1.009–1.023	<0.001
Male	0.364	0.814–1.750	0.36
Age	1.020	1.006–1.034	0.01
Body mass index (kg/m^2^)	1.028	0.976–1.082	0.30
Diabetes mellitus	2.902	1.946–4.330	<0.001
PD duration (months)	0.995	0.990–1.001	0.08
Residual urine	0.656	0.449–0.958	0.03
White blood cell count (1000/μL)	1.077	1.011–1.147	0.02
Hemoglobin (g/dL)	0.831	0.742–0.931	0.001
Platelet count (1000/μL)	1.001	0.999–1.003	0.23
AST (U/L)	1.008	0.993–1.022	0.30
ALT (U/L)	1.004	0.995–1.013	0.42
Total bilirubin (mg/dL)	0.139	0.000–178.189	0.59
Albumin (g/dL)	0.326	0.233–0.455	<0.001
Total cholesterol (mg/dL)	0.996	0.992–1.000	0.06
HDL (mg/dL)	1.002	0.981–1.024	0.86
LDL (mg/dL)	0.994	0.986–1.003	0.20
Triglyceride (mg/dL)	1.002	0.999–1.003	0.23
Sugar (mg/dL)	1.006	1.004–1.008	<0.001
HbA1c %	1.360	1.193–1.550	<0.001
Creatinine (mg/dL)	0.901	0.839–0.967	0.004
Uric acid (mg/dL)	0.953	0.816–1.113	0.54
Calcium (mg/dL)	0.830	0.691–0.997	0.05
Phosphorus (mg/dL)	0.797	0.681–0.933	0.01
iPTH (pg/mL)	1.000	0.999–1.000	0.21
Transferrin saturation	0.416	0.078–2.207	0.30
Ferritin (μg/L)	1.000	1.000–1.000	0.56
Dialysate/plasma creatinine	19.105	4.168–87.571	<0.001
Peritoneal equilibration test (%)	1.579	1.237–2.014	<0.001
Weekly CCr (normalized)	1.000	0.987–1.014	0.9
Weekly CCr (total)	1.000	0.988–1.013	0.9
Weekly CCr (PD)	1.017	1.001–1.033	0.04
Weekly CCr (renal)	0.988	0.973–1.004	0.13
Infection episodes	5.153	3.225–8.232	<0.001
Cardiovascular events	2.886	1.921–4.275	<0.001

Note:

Abbreviations: RR, relative risk; HR, hazard ratio; CI, confidence interval; HS-CRP, high-sensitivity C-reactive protein; PD, peritoneal dialysis; AST, aspartate transaminase; ALT, alanine transaminase; HDL, high-density lipoprotein; LDL, low-density lipoprotein; HbA1c, glycated hemoglobin; iPTH, intact parathyroid hormone; CCr, creatinine clearance.

**Table 7:** Relative risk of technique failure in multivariate Cox regression analysis.

		Multivariate analysis	
	RR	HR (95% CI)	P
HS-CRP (mg/L)	1.009	1.001–1.017	0.03
Diabetes mellitus	2.170	1.424–3.306	<0.001
Hemoglobin (g/dL)	0.877	0.774–0.994	0.04
Albumin (g/dL)	0.631	0.418–0.953	0.03
Dialysate/plasma creatinine	6.133	1.152–32.656	0.03
Infection episodes	4.775	2.931–7.778	<0.001
Cardiovascular events	1.896	1.228–2.927	0.004

Note:

Abbreviations: RR, relative risk; HR, hazard ratio; CI, confidence interval; HS-CRP, high-sensitivity C-reactive protein.

## Discussion

This study is a cross-sectional cohort study that enrolled 402 patients to assess the impact of serum HS-CRP levels on mortality and technique survival in PD patients. The major causes of death and technique failure in PD patient is attributed to cardiovascular disease, infection, and loss of dialysis adequacy, all of which are associated with inflammation. Our results demonstrate that HS-CRP as an inflammatory marker predicts mortality and technique survival, supporting the hypothesis that inflammation has an adverse effect on the clinical outcome. Our study demonstrates that 42% of PD patients had serum HS-CRP levels more than 5 mg/L, slightly higher than those reported in Chinese PD patients (36%) [7]. In this study, the patients in the high HS-CRP tertile have a higher mortality rate and a lower technique survival rate during the 24-month follow-up. Relatively small number of patient deaths may contribute to insignificant difference in mortality and technique survival rate between T1 (none of patients) vs T2 (4 patients). In accordance, the results of multivariate logistical regression analysis also showed that HS-CRP is an independent predictor of both mortality and technique failure.

During the past decade, CRP has emerged as a powerful predictor of mortality in dialysis patients [10], [30]–[34]. Our study also verifies the importance of HS-CRP in predicting the subsequent survival of PD patients. CRP is the most used inflammatory marker. It has been associated with the nutrition status and its markers, including serum albumin level [35], [36]. In accordance, our study also showed that serum albumin level was significantly reduced across the 3 tertiles of increasing HS-CRP. Serum albumin level is an important predictor of all-cause mortality in both PD and hemodialysis patients [37], [38]. As frequently there is a reciprocal interaction between serum albumin and CRP levels, some studies have shown that on multivariate Cox regression analysis, serum CRP level instead of serum albumin level remains significant in predicting mortality [39]. In contrast, our study showed that both serum HS-CRP and albumin levels were independent risk factors for mortality and technique failure. This discrepancy may be attributed to the low percentage (17.7%) of patients with diabetes in our study, as serum albumin level has been reported to be lower in diabetic PD patients and may become a confounding factor in studies recruiting a high population of diabetic patients. CRP has been shown to be strongly associated with diabetes in recent years [40], [41]. Oh et al. reported that diabetic PD patients had a higher serum HS-CRP level, suggesting an interrelation between diabetes and HS-CRP. Our study demonstrates that on multivariate Cox regression analysis, serum HS-CRP, albumin level, and diabetes all remain independent factors for predicting mortality and technique failure in PD patients.

Both peritoneal clearance and residual renal function are major determinants of survival in PD patients [42]–[44]. In addition, these 2 factors have been shown to be linked with serum CRP level. Some studies reported that a reduction of residual renal function and peritoneal clearance led to an elevated serum CRP level [24], [25], [45]–[47], whereas other studies did not find this correlation [19], [22], [29], [48]. There is therefore a controversy about whether CRP predicts the clinical outcome of PD patients independent of peritoneal clearance and residual renal function. Our study demonstrated that peritoneal clearance was decreased across the 3 tertiles of increasing HS-CRP, whereas there was no significant difference in the CCr of residual renal function across the 3 tertiles. On multivariate logistic regression analysis, serum HS-CRP level instead of peritoneal clearance and residual renal function was a negative predictive marker for both mortality and technique failure. The CANUSA study showed that there was a 12% decrease in the relative risk (RR) of death (RR, 0.88; 95% confidence interval [CI], 0.83 to 0.94) for each 5 L/wk per 1.73 m2 increment in glomerular filtration rate [49]. Compared with the patients in the CANUSA study, our patients had less residual renal function (37.7 L/wk in CANUSA study *vs.* 8.9 L/wk in our study). Thus, our study might underestimate the contribution of residual renal function to mortality and technique failure.

Technique failure in PD patients is frequently caused by peritonitis or high peritoneal membrane solute transport [42], [43], [48], [50], [51]. Our data showed that the patients in the high HS-CRP tertile had a higher value of D/P_Cr_ and a larger proportion of high transporters than those in the other tertiles. In addition, despite having no role in predicting mortality, a high D/P_Cr_ value is indeed a high risk factor for technique failure. Furthermore, high HS-CRP level predicted subsequent technique failure independent of D/P_Cr_. Being a high transporter has been shown to be a significant risk factor for PD failure in a large-scale study [48], and associated with a trend to higher technique failure in a meta-analysis report [43]. In addition, Fine et al. have demonstrated that being a high transporter predicts an increase in serum CRP level [45]. Whether the association between a high transporter status and serum HS-CRP level is a causal relation or an epiphenomenon remains elusive.

Cardiovascular events are the most common cause of death in dialysis patients [52], [53]. It may be related to uremic toxins, volume status, vascular calcification, anemia, hypoalbuminemia, and chronic inflammation [52], [54], [55]. An elevated CRP level has been reported to be an independent predictor of myocardial infarction and cardiovascular mortality in PD patients [7], [15], [19], [20]. Our study further demonstrated that although the incidence of cardiovascular events was significantly increased across the increasing HS-CRP tertiles, both HS-CRP and cardiovascular events were independent predictive markers for all-cause mortality on multivariate Cox regression analysis.

Our study also verified some well-established factors associated with HS-CRP (Table 1), including age, DM, BMI, existence of residual urine, white blood cell count, hemoglobin, albumin, HDL, triglycerides, fasting sugar, HbA1c level, and transferrin saturation. Among these factors, it is of interest that fasting sugar and HbA1c levels were significantly increased across the 3 tertiles in non-diabetic patients, whereas it lost statistical significance in diabetic patients. In addition to blood sugar control, chronic inflammation and oxidant stress have been found to be elevated in diabetic dialysis patients. Our finding may suggest that other factors, such as oxidative stress and chronic inflammation, in diabetic PD patients may surpass the influence of blood sugar control on serum HS-CRP level, whereas blood sugar level is still associated with the elevated serum HS-CRP level in nondiabetic PD patients. Nevertheless, except HS-CRP, age, DM, and hypoalbuminemia, all of these factors, including fasting sugar and HbA1c level, failed to predict mortality and technique failure on multivariate logistical regression analysis.

This study has several limitations. First, this study was restricted to a single-center observation. Second, this study is cross-sectional and includes prevalent patients. As HS-CRP is associated with high mortality in PD patients, this study enrolled only prevalent patients and may have underestimated the impact of HS-CRP on mortality owing to censoring bias. Nevertheless, the large number of PD patients and the extensive analysis of possible parameters that can affect clinical outcome in this study reduce these limitations.

In conclusion, the present study shows the importance of HS-CRP in predicting the 2-year mortality and technique survival in PD patients independent of age, diabetes, hypoalbuminemia, and the occurrence of cardiovascular events.
